# Mitigation of Humic Acid Inhibition in Anaerobic Digestion of Cellulose by Addition of Various Salts

**DOI:** 10.3390/bioengineering2020054

**Published:** 2015-03-25

**Authors:** Samet Azman, Ahmad F. Khadem, Grietje Zeeman, Jules B. van Lier, Caroline M. Plugge

**Affiliations:** 1 Laboratory of Microbiology, Wageningen University, Dreijenplein 10, 6703 HB Wageningen, The Netherlands; E-Mails: ahmad.khadem@wur.nl (A.F.K.); caroline.plugge@wur.nl (C.M.P.); 2 Sub-Department of Environmental Biotechnology, Wageningen University, 6700 AA Wageningen, The Netherlands; E-Mail: grietje.zeeman@wur.nl; 3 Section Sanitary Engineering, Department of Water Management, Faculty of Civil Engineering and Geosciences, Delft University of Technology, 2600 GA Delft, The Netherlands; E-Mail: J.B.vanLier@tudelft.nl

**Keywords:** cellulose, anaerobic digestion, humic acid, hydrolysis, inhibition, mitigation, salt addition, cations

## Abstract

Humic compounds are inhibitory to the anaerobic hydrolysis of cellulosic biomass. In this study, the impact of salt addition to mitigate the inhibitory effects of humic compounds was investigated. The experiment was conducted using batch tests to monitor the anaerobic hydrolysis of cellulose in the presence of humic acid. Sodium, potassium, calcium, magnesium and iron salts were tested separately for their efficiency to mitigate humic acid inhibition. All experiments were done under mesophilic conditions (30 °C) and at pH 7. Methane production was monitored online, using the Automatic Methane Potential Test System. Methane production, soluble chemical oxygen demand and volatile fatty acid content of the samples were measured to calculate the hydrolysis efficiencies. Addition of magnesium, calcium and iron salts clearly mitigated the inhibitory effects of humic acid and hydrolysis efficiencies reached up to 75%, 65% and 72%, respectively, which were similar to control experiments. Conversely, potassium and sodium salts addition did not mitigate the inhibition and hydrolysis efficiencies were found to be less than 40%. Mitigation of humic acid inhibition via salt addition was also validated by inductively coupled plasma atomic emission spectroscopy analyses, which showed the binding capacity of different cations to humic acid.

## 1. Introduction

Lignocellulosic biomass has been thoroughly studied for its energy potential, since there is an extensive effort to replace fossil fuels with renewable energy sources [1]. Although, renewable energy from biomass can be produced by several processes, anaerobic digestion is one of the widely used processes to convert the chemically enclosed energy in the biomass to biogas [2]. However, currently available technologies for anaerobic biomass digestion are not always efficient in converting biomass into biogas. Low biogas production within biomass digesters is mainly related to low hydrolysis rates and limited substrate biodegradability [3,4,5]. Hydrolysis is the first step of anaerobic digestion in which complex molecules are converted to soluble monomers or/and oligomers. Hydrolysis is often considered as the rate limiting step in anaerobic digestion of biomass [6]. This rate limitation phenomenon can be explained by the encrustation of biomass by lignin, and the presence of humic compounds [7,8,9,10].

Humic acids (HA) are complex mixtures of different organic molecules that are produced during decay and transformation of organic matter. HA are resistant to biodegradation but they can react physically and chemically with several compounds due to their weak polyelectrolyte behaviour at the same time. Because of the weak polyelectrolyte behaviour, HA can dissociate in aqueous solutions, and make several compounds partially charged. These properties make HA important components of soil, lake/sea sediments and anaerobic digester environments in which they affect the physicochemical properties such as; bio-availability of enzymes, metals and macro/micro nutrients, and biological processes [11,12]. In an anaerobic digester environment, abundance and composition of HA mainly depend on the type of feed [10]. Although, HA content within anaerobic digesters are not well defined in the literature, HA concentrations can reach up to 1.5% w/w of total solids in the treatment sludge, manure and maize [10,13,14].

Fractions of HA can affect the biodegradation of biomass during anaerobic digestion since they strongly inhibit cellulose hydrolysis [15,16]. Although the exact mechanism of HA inhibition on hydrolysis is not known, binding properties of HA to hydrolytic enzymes are proposed for such an inhibition [15]. Fernandes *et al.* [15] hypothesized that binding of hydrolytic enzymes to HA lower the availability of enzymatic activity for cellulose hydrolysis. Fernandes *et al.* [15] observed strong inhibitory effects of HA on cellulose hydrolysis in batch tests. Thus, there is a need to reverse the inhibitory effects of HA on hydrolysis to improve cellulolytic biomass digestion. Consequently, to eliminate HA inhibition on cellulolytic biomass digestion, two approaches can be followed: (i) removal of the HA from the related environment and (ii) mitigation of the inhibitory effects by adding compounds that can reduce the binding capacity of HA. Removal of HA from aquatic environments has been successfully achieved by adding coagulants and flocculants to contaminated sites [17,18]. Utilization of ion exchange resins was also reported as a successful method to remove HA from groundwater [19]. However, the increased solids content of biomass hampers the application of the aforementioned methods in anaerobic digesters. Alternatively, removal of HA by extraction methods can be considered as a solution in anaerobic digesters. A recent study showed that the extraction of HA via alkali pre-treatment methods from primary sludge increased the total methane yield by 50% [14]. Although removal methods can be successful in lab-scale applications, their economic and practical feasibility for large scale applications is highly questionable. Therefore, mitigation strategies seem to have higher potential to overcome HA inhibition on hydrolysis during organic matter digestion, as mentioned in a few literature studies. In 1985, Brons *et al.* [16] showed that the addition of Ca^2+^ cations reversed the inhibitory effects of humate on potato protein hydrolysis. More recently, Fernandes *et al.* [15] proposed that the addition of excess amounts of hydrolytic enzymes may help to overcome HA inhibition. Although some methods were suggested to mitigate hydrolysis inhibition, detailed information was not available about mitigation of HA inhibition on anaerobic cellulose degradation.

In our present study, we aim to show the mitigation of HA inhibition on anaerobic cellulose digestion by adding several salts. Following the discussions of Fernandes *et al.* [15], we hypothesized that reducing active enzyme binding sites of HA with cations may reverse the hydrolysis inhibition and subsequently increase the methane production. In this scope, batch tests were set-up to monitor anaerobic digestion of cellulose in the presence of HA and salt addition was tested to find successful candidates to mitigate HA inhibition. During the experiment, hydrolysis efficiencies, methane yields and corresponding methane production rates were monitored with Chemical Oxygen Demand (COD) COD and Volatile Fatty Acid (VFA) analyses, to evaluate the utilization potential of Na^+^, K^+^, Ca^2+^, Mg^2+^ and Fe^3+^ salts in mitigation of HA inhibition.

## 2. Experimental Section

### 2.1. Experimental Set-Up

Batch incubations were performed in 500 mL glass bottles, which contained 400 mL liquid anaerobic medium with the following composition in µM: 5000 Na_2_HPO_4_, 5000 KH_2_PO_4_, 5600 NH_4_Cl, 680 CaCl_2_, 600 MgCl_2_, 5000 NaCl, 7.5 FeCl_2_, 1 H_3_BO_3_, 50 HCl, 0.5 ZnCl_2_, 0.5 MnCl_2_, 0.5 CoCl_2_, 0.1 NiCl_2_, 0.1 Na_2_SeO_3_, 0.1 Na_2_WO_4_, 0.1 Na_2_MoO_4_ and vitamins (µg·L^−1^); 0.02 biotin, 0.2 nicotinic acid, 0.5 pyridoxine, 0.1 riboflavin, 0.2 thiamin, 0.1 cyanocobalamin, 0.1 p-aminobenzoic acid, 0.1 pantothenic acid. The bottles were inoculated with granular, methanogenic sludge from a full-scale Up-flow Anaerobic Sludge Blanket reactor (Eerbeek, the Netherlands). The reactor is treating pulp and paper industry waste water. Avicel PH-101 (Fluka) was chosen as model substrate at 1 g·L^−1^ COD (unless otherwise stated) and the ratio between substrate and microorganisms was set to 0.8 (g·VS/g VSS) to obtain enough carbon for the inoculum [20]. All bottles were flushed with nitrogen gas prior to the start of the experiment.

The experiment bottles were set up in duplicates, including blank controls, positive controls, inhibition groups and salt addition groups. To the blank control, no carbon source was added, to determine the endogenous activity of the inoculum. In the positive controls, Avicel was added as a carbon source to determine net Avicel biodegradation. In the inhibition groups, 5 g·L^−1^ HA (Sigma-Aldrich; CAS Number: 68131-04-4) was added to create an inhibitory environment for Avicel biodegradation [15]. In salt addition controls, 5 mM of NaCl, KCl, CaCl_2_, MgCl_2_ and FeCl_3_ were separately added to HA and Avicel containing experimental bottles to determine the effects of salt additions on HA inhibition and Avicel biodegradation. Table 1 summarizes the experimental groups that were used in the whole experiment. The experiment was carried out for 14 days, at 30 °C. pH was set to 7 at the beginning of the experiment and all the experiments were conducted in between pH 6.8 and pH 7. Intermittent stirring was applied (60 s on and 60 s off at 90 rpm) to obtain efficient mixing in the experimental bottles. The first sampling was done immediately after all bottles were prepared. Then, sampling was done at four different times after 0, 96, 168 and 288 h. During the experiment, biogas production was monitored online by using Automatic Methane Potential Test System II (AMPTS II, Lund, Sweden). Soluble carbon content of the samples was determined by Chemical Oxygen Demand (COD) and Volatile Fatty Acid (VFA) analyses. Salt addition experiment bottles, were also analyzed by Ion chromatography, to determine the binding capacity of Na^+^, K^+^, Ca^2+^, Mg^2+^ and Fe^3+^ to HA.

**Table 1:** Summary of the experimental set-up and abbreviations that are used in the text.

Experimental Group	Abbreviation	Avicel g/L COD	HA g/L	Added Salt (mM)
**Blank** (negative control)	**-**	0	0	0
**Avicel** (positive control)	**C**	1	0	0
**Avicel + HA** (inhibition group)	**I**	1	5	0
**Avicel + HA + KCL** (Salt addition group)	**K**	1	5	5
**Avicel + HA + NaCl** (Salt addition group)	**Na**	1	5	5
**Avicel + HA + CaCl_2_** (Salt addition group)	**Ca**	1	5	5
**Avicel + HA + MgCl_2_** (Salt addition group)	**Mg**	1	5	5
**Avicel + HA + FeCl_3_** (Salt addition group)	**Fe**	1	5	5

### 2.2. Monitoring Methane Production

Biogas production of the experimental groups, was measured online by the AMPTS II (Bioprocess Control, Lund, Sweden), and according to the protocol described by Badshah and coworkers [21]. Hourly recorded results were used to determine methane production and methane production rates. All measured gas volumes were normalized to standard temperature and pressure conditions (273 K, 1 atmospheric pressure and zero moisture content) and the results were corrected for the recorded values in the negative controls.

### 2.3. Analytical Methods

Liquid samples were centrifuged (13,000× rpm, room temperature, 10 min), and the obtained supernatant was filtered through a polypropylene filter (Ø 0.45 µm). Supernatants that contained HA were first acidified with 1 M H_2_SO_4_ to pH 3 and subsequently centrifuged to remove HA. Acidified samples were neutralized to pH 7, prior to filtration. Samples without HA and VFA standards were treated using the same procedure. The filtered supernatant, was then analyzed for VFA using a High Liquid Pressure Chromatography (Thermo Scientific Spectra System, HPLC), equipped with a Varian MetaCarb 67H column (300 mm × 6.5 mm), which was connected to a UV and refractive index detector (Middelburg, The Netherlands). The mobile phase and internal standard were 10 mM Sulfuric acid and arabinose, respectively. The eluent had a flow of 0.8 mL/min. Data analyses, were performed with the ChromQuest (Thermo Scientific, Waltham, MA, USA) and Chromeleon software (Thermo Scientific, Waltham, MA, USA).

Soluble COD analyses were done with COD cell kits (Spectroquant, 14,541) from Merck (Darmstadt, Germany), according to the manufacturer’s instructions.

The efficiency of hydrolysis, acidogenesis and methanogenesis was calculated using Equations (1)–(3), in which H is the hydrolysis efficiency (%) corrected for the soluble COD fraction at the start of the experiment, A is the acidogenesis efficiency (%) and M is the methanogenesis efficiency (%); CODm, *t* = x is methane expressed as COD (*t* = time; x = sampling time). CODs, *t* = x is the soluble COD at *t* = x, CODv, *t* = x is the VFAs at t = x and CODtotal, *t* = 0 is the total COD added at the beginning of each experiment.

(1) $$H\left(\%\right)=\frac{CODm,\;t\;=\;x\;+\;CODs,\;t=x\;-\;CODs,t=0\;}{COD\;total,\;t=0}\times 100$$

(2) $$A\;(\%)\;=\frac{CODm,\;t\;=\;x\;+CODv,\;t=x\;}{COD\;total,\;t=0}\times 100$$

(3) $$M\;\left(\%\right)=\frac{CODm,\;\;t=x\;}{COD\;total,\;\;t=0}\times 100$$

Solubilized substrate, at *t* = 0 was removed from the equation to assess specifically the hydrolysis of the particulate matter. Equation (1), which calculates the actual hydrolysis efficiency (H), was used for this assessment.

Volatile solid content of the substrates (VS), Volatile Suspended Solids (VSS) content of the inoculum and pH values were determined, using standard methods [22].

For the ion chromatography analyses (Na^+^, K^+^, Ca^2+^, Mg^2+^ and Fe^3+^ were measured), liquid samples were centrifuged (10,000× *g*, RT, 5 min), and were subsequently measured (with technical triplicates of duplicate samples) by Inductively Coupled Plasma Atomic Emission Spectrometry (ICP-AES) using a Vista MPX ICP-AES instrument.

## 3. Results and Discussion

### 3.1. Hydrolysis, Acidogenesis and Methanogenesis Efficiencies

**Figure 1:**
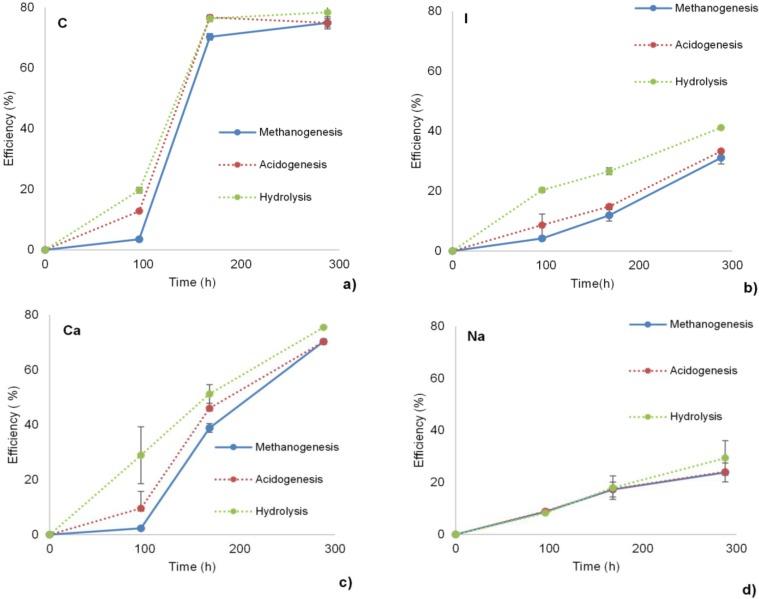

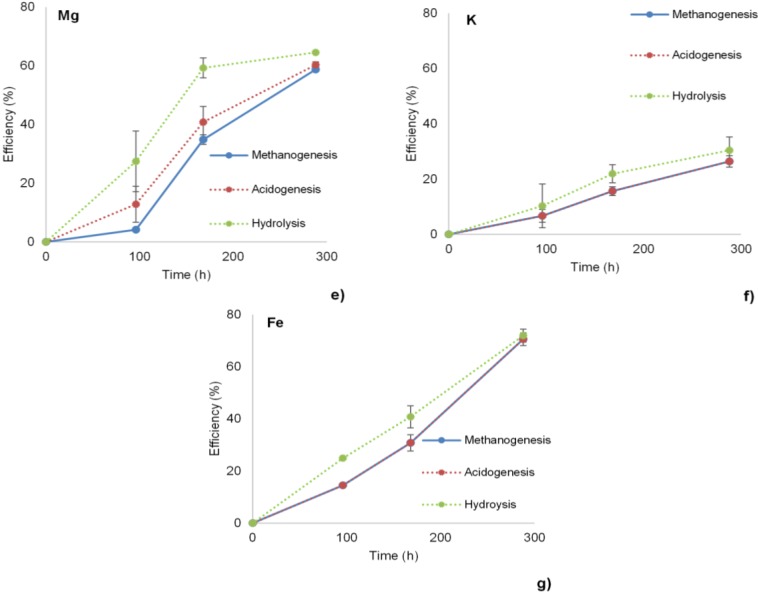
Salt addition experiments, namely efficiency (%) of methanogenesis, acidogenesis and hydrolysis over time. Results of each experimental group is illustrated with; (**a**) positive control (C), (**b**) inhibition control (I), salt addition experiment group of; (**c**) calcium (Ca), (**d**) sodium (Na), (**e**) magnesium (Mg), (**f**) potassium (K) and (**g**) iron (Fe)salts. (Error bars show the standard deviation between measurements, *n* = 2).

Hydrolysis, acidogenesis and methanogenesis efficiencies of the experimental groups were calculated with the formulas that were given in analytical methods section (Figure 1). During the experiments, VFA production was detected in most of the samples. The amount of detected VFAs was relatively low, *i.e.*, less than 20% of the overall COD or VFAs were not detected at all, likely due to the rapid conversion of VFAs to methane. Acetate and propionate were found as the dominant VFAs. Results from the positive controls showed that hydrolysis was almost completed within the first 170 h of the experiment. Hydrolysis and methanogenesis efficiencies were calculated as 78% which are commonly found for crystalline cellulose (Avicel) with the selected inoculum concentrations [23]. Complete degradation of VFAs accompanied by hydrolysis in positive controls indicated efficient digestion profiles for the cellulose (Figure 1a). In the inhibition experimental group, hydrolysis efficiency was reduced by 50%, compared to the positive controls which showed the HA inhibition (Figure 1b). The hydrolysis efficiency for the inhibition groups was 40% higher than previously reported [15]. The main reason for the higher hydrolysis efficiency might be related to the type of HA (HA extracted from maize and manure) that was used. It is known that different types of HA has different effects due to the source of HA source and the extraction methods used [10]. On the other hand, VFA accumulation was observed in the inhibition groups, which indicated that HA possibly inhibited methanogenesis. The observed negative effects of HA on methanogenesis was previously reported by Brons and co-workers. They observed a significant delay in the methane production during potato protein digestion, in the presence of humate [16].

In the salt addition groups, calcium, magnesium and iron salts mitigated the inhibitory effect of HA on hydrolysis. In the Ca, Mg and Fe salt addition groups, hydrolysis efficiencies were 75%, 65% and 72%, respectively after 300 h incubation (Figure 1c–e). In addition to hydrolysis efficiencies, acidogenesis efficiencies were slightly higher than methanogenesis efficiencies that indicated a delay in methanogenesis which was recovered at the end of the experiment. Recovered hydrolysis, acidogenesis and methanogenesis, compared to positive controls, at the end of the experiment revealed the positive effect of addition of Ca, Mg and Fe salt. The overall results showed that addition of calcium, magnesium and iron salts mitigated hydrolysis inhibition, most probably, by shielding or attachment to the active binding sites of the HA. Apparently, reducing the number of active binding sites prevented scavenging of hydrolytic enzymes from the liquid media that consequently improved the cellulose hydrolysis and therefore methanogenesis [15].

On the other hand, sodium and potassium salts did not mitigate the hydrolysis inhibition (Figure 1f,g, respectively). In these salt addition groups, hydrolysis efficiency was 30% which was slightly lower than the hydrolysis efficiency of inhibition groups even though they were expected to show similar results. This may indicate the possible inhibition caused by the high concentration of monovalent sodium and potassium cations [24]. Thus, sodium and potassium salts were not effective to diminish the inhibitory effect of HA.

### 3.2. Methane Yield and Methane Production Rates

**Figure 2:**
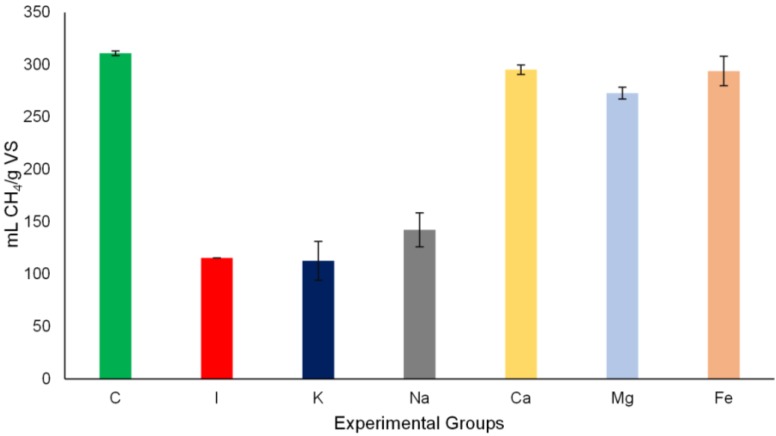
Methane yield (mL CH_4_/g VS) of the positive control (C), inhibition control (I) and salt addition controls; with calcium (Ca), magnesium (Mg), iron (Fe), sodium (Na) and potassium (K). Yields were calculated at the end of the anaerobic degradation tests (14 days). (Error bars show the standard deviation between measurements, *n* = 2).

The effects of HA on anaerobic digestion of cellulose (Avicel) and strategies to overcome HA inhibition were evaluated in terms of methane yield and methane production rates. In Figure 2, the overall methane yield achieved for the cellulose digestion within the experimental groups is shown. Avicel degradation yielded 310 mL CH_4_/g VS at the end of the experiment which was previously found for this type of cellulose [25]. However, HA addition decreased the methane yield three fold, which showed again the strong inhibitory effect of HA on the anaerobic digestion of cellulose. Since HA inhibition was mitigated with the addition of calcium, magnesium and iron salts, consequently, methane yield was recovered to 295 mL, 273 mL, and 294 mL CH_4_/g VS, respectively. The sodium and potassium salt additions did not improve the methane yield.

**Figure 3:**
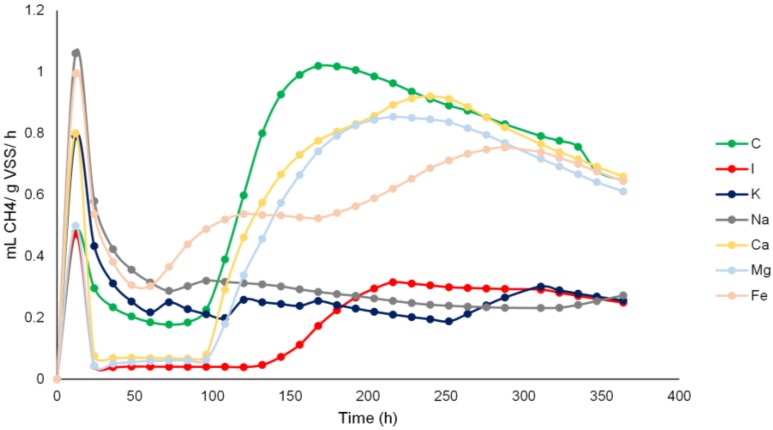
Maximum methane production rates of positive control (C), inhibition group (I) and salt addition groups; of calcium (Ca), magnesium (Mg), iron (Fe), sodium (Na) and potassium (K).

Methane production rates were assessed in the positive controls, inhibition groups and salt addition groups to determine the corresponding maximum methane production rates (Figure 3). The first activity peaks were observed in the first 50 h, which can be attributed to the conversion of easily degradable substrates, such as residual glucose in Avicel powder (Figure 3). The second activity peaks were observed between 100 and 300 h, and were related with cellulose hydrolysis (Figure 2). No clear second peaks were found in the Na and K salt addition groups, indicative of low hydrolysis activity. The positive controls showed the highest methane production rate ((1 mL CH_4_/g VSS)/h, on hour 168). In the presence of HA, maximum methane production rate was 0.3 mL CH_4_/g VSS/h, showing once more the inhibitory effect of HA. In addition to methane yields, Ca, Mg and Fe salts increased the maximum methane production rates to 0.92 mL, 0.85 mL and 0.75 mL CH_4_/g VSS/h, respectively. However, maximum production rates in salt addition groups were still lower than in the positive controls, and it took a longer amount of time to reach the maximum activity.

### 3.3. Effects of Salt Addition

The liquid phase of all experimental groups was analyzed by ICP-AES for Na, K, Ca, Mg and Fe to validate the interaction of HA with the respective cations. The cation concentrations were analysed at the beginning (initial = i) and at the end (final = f) of the experiment (Table 2). HA addition to the inhibition groups introduced a significant amount of sodium (approximately 300 mg/L) and a small amount of calcium and iron to the anaerobic media, when compared with the positive controls (Table 2). Although an excess of sodium could potentially inhibit anaerobic digestion, sodium in HA was still 10-fold lower than inhibitory sodium concentrations that were previously reported [24,26,27]. Moreover, the presence of the other cations such as potassium, magnesium and calcium, are likely to show antagonistic effects to sodium inhibition [24].

**Table 2:** Cation concentrations of the experimental groups in the beginning (initial = i) and at the end (final = f) of the experiment. Results of each experimental group abbreviated with; positive control (C), inhibition groups (I), salt addition groups of calcium (Ca), magnesium (Mg), iron (Fe), sodium (Na) and potassium (K).

	Cations (mg/L)				
Samples	K^+^	Na^+^	Ca^2+^	Mg^2+^	Fe^2+^ + Fe^3+^
**C-i**	116.03	217.4	24.81	12.947	<0.001
**C-f**	85.6 ± 0.44	160.71 ± 0.26	14.51 ± 0.38	10.42 ± 0.01	<0.001
**I-i**	166.11	525.97	85.33	13.54	32.05
**I-f**	137.47 ± 4.03	523.22 ± 17.8	91.58 ± 1.35	13.47 ± 0.55	32.85 ± 1.71
**K-i**	306.515	514.01	60.165	12.168	23.437
**K-f**	298.67 ± 0.29	494.31 ± 0.28	61.83 ± 3.2	12.80 ± 0.78	31.31 ± 2.9
**Na-i**	141.59	717.24	80.57	13.1	25.46
**Na-f**	142.1 ± 0.26	679.24 ± 11	82.81 ± 0.12	14.44 ± 0.4	37.35 ± 0.4
**Ca-i**	128.63	501.2	**285.08**	6.48	**32.05**
**Ca-f**	123.71 ± 1.71	470.98 ± 9.16	**32.63 ± 0.93**	4.89 ± 0.11	**0.27 ± 0.01**
**Mg-i**	127.87	517.42	**99.331**	134.958	**32.047**
**Mg-fi**	107.3 ± 2.24	424.56 ± 8.56	**9.51 ± 0.24**	117.31 ± 0.3	**<0.001**
**Fe-i**	124.95	688.05	68.40	10.84	**347.38**
**Fe-f**	145.39 ± 0.52	643.30 ± 3.69	89.59 ± 0.04	15.35 ± 0.21	**55.44 ± 1.50**

In the inhibition groups, no significant changes were found between initial and final concentration of cations. However, significant changes in cation concentrations were found within the salt addition groups of Ca, Mg and Fe. In the Ca salt addition groups, about 90% of the calcium and 99% of iron cations were removed from the liquid phase, whereas potassium, sodium and magnesium concentrations remained constant. Interestingly, in the Mg group, only 10% of the externally added magnesium was removed from the liquid phase. Magnesium chloride addition apparently promoted the binding of calcium and iron cations to HA. Therefore, utilization of the magnesium salts may be more feasible due to the possibility of recycling unbounded magnesium cations in larger scale applications. In the Fe group, 85% of the iron cations were removed from the liquid phase, while no significant changes were observed for the other cations.

The removal of cations from the liquid phase seems to be directly related with the interaction of cations with HA. The proposed interaction is more likely in terms of ionic binding with the formation of HA-cation complexes. The cation binding and complexation with HA can be explained by the model that was described by Tipping [28]. According to this model, binding of cations to HA takes place at discrete sites of binding domains of HA by electrostatic attraction. Also, counter ion accumulation in the environment promotes non-specific binding of cations to HA. Therefore, the strength of the binding depends on the valence of the cations. In this respect, divalent or trivalent cations have more affinity for HA [28]. The results from this study also validated the aforementioned theory [12,28]. HA formed precipitates with calcium and magnesium cations whereas sodium and potassium did not form precipitates. In the Fe groups, the precipitation was not observed even though iron has a higher valence than the other cations. Loose binding between iron and HA can be explained by the chemical reduction of Fe^3+^ to Fe^2+^ in anaerobic environments [29]. Because of this reduction, strong Fe^3+^-HA bonds might be converted to weaker Fe^2+^-HA binding [12]. The other weak binding, observed with sodium and potassium, was not very surprising, since these cations are recognized as deflocculating agents [30]. Therefore, they probably prevented binding via increasing the repulsion between the HA and cations.

## 4. Conclusions

Methane potential and hydrolysis efficiencies of cellulose are noticeably decreased in the presence of HA In the present study, it was demonstrated that it was possible to reduce the active sites of HA with salt addition, mitigating the inhibitory effects of HA on the hydrolysis and consequently on the methane yields. Compared to HA inhibited groups, calcium, magnesium and iron salt addition increased the methane yields by 60% and increased the hydrolysis efficiencies by 30%, whereas sodium and potassium salts addition did not mitigate HA inhibition. Even though calcium and magnesium mitigated the HA inhibition, the affinity of each cation to HA was not the same. Binding strength of the cations to HA increased in order K^+^ = Na^+^ < Mg^2+^ < Fe^3+^ (Fe^2+^) < Ca^2+^. Although the proposed method was successful in batch tests, it is worth testing the effects of calcium or magnesium salts to continuously fed anaerobic bioreactors, treating high solid content residues.
